# Posterior pedicle screw fixation for complex atlantoaxial fractures with atlanto-dental interval of ≥5 mm or C2-C3 angulation of ≥11°

**DOI:** 10.1186/s13018-014-0104-5

**Published:** 2014-11-19

**Authors:** Lei Wang, Chao Liu, Qinghua Zhao, Jiwei Tian

**Affiliations:** Department of Orthopaedics, Shanghai Jiaotong University Affiliated First People’s Hospital, 100 Haining Road, Shanghai, 200080 China; Shanghai Jiao Tong University School of Medicine, Shanghai, 200025 China

**Keywords:** Complex atlantoaxial fractures, Surgical treatment, Screw fixation

## Abstract

**Objective:**

Previous studies have demonstrated that the posterior pedicle screw fixation is an effective and safe method to treat atlantoaxial fractures. However, no report focuses on only the complex atlantoaxial fractures with atlanto-dental interval (ADI) of ≥5 mm or C2-C3 angulation of ≥11°.

**Methods:**

This study was to retrospectively evaluate the outcome of 15 patients (six females and nine males; age, 27–55 years) who underwent posterior pedicle screw fixation for the above complex atlantoaxial fractures between July 2006 and March 2011. Fracture combinations included three Jefferson-type II odontoid, four anterior ring-type II odontoid, two posterior ring-type II odontoid, one lateral mass-type II odontoid, one Jefferson-hangman’s fracture, three anterior ring-hangman’s fracture, and one lateral mass-hangman’s fracture. Fracture healing and bone fusion were determined on X-ray scan. Upper limbs, lower limbs, and sphincter functions were assessed using the Japanese Orthopaedic Association (JOA) score. The Frankel grading system was used to determine the neurological situation.

**Results:**

The mean operative time, blood loss, and hospital stays were 108.9 ± 25.8 min, 508.0 ± 209.6 ml, and 13.3 ± 2.0 days. Fracture healing and graft fusion were obtained in all patients within 9 months. The ADI or C2-C3 angulation was reduced to ≤5 mm or ≤11°. The JOA score was significantly improved from 7.27 ± 1.10 preoperatively to 15.7 ± 2.1 postoperatively (*P* <0.001), with 88.1 ± 18.3% recovery rate and 93.3% excellent and good rate. The neurological situation was improved in all patients by at least 1 grade in the Frankel scale. After a mean of 36.5 months of follow-up (range, 18 to 58 months), no operative complications (spinal cord injury, vertebral artery injury, or cerebrospinal fluid leakage) were observed.

**Conclusion:**

Posterior pedicle screw fixation is a reliable, effective, and minimally invasive procedure for patients suffering from complex atlantoaxial fractures.

## Introduction

Complex atlantoaxial fracture is a relatively rare clinical injury, accounting for 3% of all acute cervical spine injuries, 43% of atlas fractures, and 16% of axis fractures [1]. Atlas-axis fracture combination types include C1-type II odontoid fractures, C1-miscellaneous axis fractures, C1-type III odontoid fractures, and C1-hangman’s fractures [2]. According to the previous guidelines [3,4], patients with C1-stable type II odontoid fractures, C1-miscellaneous axis body fractures, C1-type III odontoid fractures, and stable C1-hangman’s fractures can be successfully treated with the use of a halo or collar immobilization device. However, a C1-type II odontoid fracture with an atlanto-dental interval (ADI) of ≥5 mm and a C1-hangman’s fracture with a C2-C3 angulation of ≥11° should be considered for early surgical treatment.

Traditionally, atlantoaxial instability can be treated by the posterior atlantoaxial short segment fixation and fusion, including the Gallie wire [5], Brooks wire [6], interlaminar clamps [7], and transarticular screw [8-10]. However, the use of these methods also results in a high incidence of internal fixation loosening or breakage, bone nonunion, and other complications. In 2001, Harms and Melcher [11] reported the stabilization of posterior atlantoaxial fractures by inserting screws in the C1 lateral mass and in the pedicle of C2. Subsequently, several studies have used this approach and demonstrated that the posterior pedicle screw fixation is an effective and safe method to treat the atlantoaxial fractures, achieving 100% fusion rate and no vertebral artery or spinal cord injury complications [11-15]. Using the same surgery strategy, this study aimed to retrospectively evaluate the treatment outcome in 15 Chinese patients who all suffered C1-type II odontoid fracture with ADI of ≥5 mm or C1-hangman’s fracture with C2-C3 angulation of ≥11°. To our knowledge, there was no report focusing on only these fractures.

## Materials and methods

### Patients

From July 2006 to March 2011, 15 cases (nine males and six females; ranged 27–55 years old, mean age of 41.3 ± 9.5) suffered fractures of the atlantoaxial complex and underwent C1-C2 pedicle screw fixation in our hospital. Fracture combinations included ten C1-type II odontoid and five C1-hangman’s fractures. The C1 fractures consisted of Jefferson, anterior ring, posterior ring, and one lateral mass fractures. Therefore, fracture combinations included three Jefferson-type II odontoid, four anterior ring-type II odontoid, two posterior ring-type II odontoid, one lateral mass-type II odontoid, one Jefferson-hangman’s fracture, three anterior ring-hangman’s fracture, and one lateral mass-hangman’s fracture (Table 1). The injury mechanisms were traffic accidents in nine cases, falling from a height in five cases, and falling down in one case. All the 15 patients showed occipito-cervical pain and limited neck movement. Besides, five patients had difficulty in sensation, movement, and reflex in the limbs. Anteroposterior, lateral, and open-mouth (atlantoaxial) X-rays, computed tomography (CT) scanning, three-dimensional (3D-CT) scanning, and magnetic resonance imaging (MRI) were used to diagnose atlantoaxial complex fractures and confirm the vertebral artery or spinal cord injury. Fifteen patients manifested nerve injury (Frankel grade C in eight cases and grade D in seven cases) [16]. Due to the retrospective nature of the study, no approvals of the patient or the local ethics committee were necessary.

**Table 1:** **Fifteen complex atlantoaxial fractures undergoing posterior pedicle screw fixation**

**Case**	**Sex/age**	**Dickman type**	**Injury mechanism**	**Pre-ADI (mm)**	**Pre-C2/C3 angulation (°)**	**Pre-nerve injury**	**Time to surgery (day)**	**Time of surgery (min)**	**Blood loss (ml)**	**Hospital stay (day)**	**Fusion time (month)**	**Fracture healing (month)**	**Post-nerve injury**	**Post-ADI (mm)**	**Pre-C2/C3 angulation (°)**	**Pre-JOA**	**Post-JOA**	**Recovery rate (%)**	**Follow-up**
1	M/38	C1 (posterior ring) -II odontoid fractures	Falling from a height	5	7	C	3	120	300	14	6	4.5	E	3	5	7	16	90	36
2	M/30	C1 (Jefferson) -hangman’s fractures	Traffic accidents	4	27	D	5	78	460	12	6	6	E	3	11	8	16	88.9	48
3	M/44	C1 (Jefferson) -II odontoid fractures	Traffic accidents	7	7	C	14	132	700	12	6	5	E	3	3	7	17	100	20
4	M/55	C1 (anterior ring) -hangman’s fractures	Falling down	4	19	D	2	90	260	12	6	6	E	3	7	8	16	88.9	56
5	M/46	C1 (anterior ring) -hangman’s fractures	Traffic accidents	4	15	C	5	100	800	14	6	6	E	3	9	7	17	100	52
6	M/55	C1 (anterior ring) -hangman’s fractures	Falling from a height	4	30	D	7	110	320	12	9	9	E	4	9	7	17	100	24
7	M/46	C1 (anterior ring) -II odontoid fractures	Falling from a height	5	5	D	5	150	900	16	9	9	E	2	3	7	16	90	20
8	M/27	C1 (lateral mass) -II odontoid fractures	Traffic accidents	5	3	C	10	84	520	12	6	3	E	3	3	8	17	100	48
9	M/38	C1 (lateral mass) -hangman’s fractures	Traffic accidents	5	13	D	14	96	280	10	9	4.5	E	3	5	10	17	100	58
10	F/48	C1 (Jefferson) -II odontoid fractures	Traffic accidents	7	5	C	3	160	720	14	6	6	E	4	3	8	16	88.9	18
11	F/48	C1 (anterior ring) -II odontoid fractures	Traffic accidents	5	7	D	7	68	280	14	6	6	E	3	3	7	17	100	48
12	F/28	C1 (Jefferson) -II odontoid fractures	Falling from a height	9	7	C	3	106	410	14	6	6	E	3	3	7	16	90	24
13	F/50	C1 (anterior ring) -II odontoid fractures	Traffic accidents	6	5	D	3	114	560	14	9	9	E	3	3	6	16	90.9	24
14	F/30	C1 (posterior ring) -II odontoid fractures	Falling from a height	6	7	C	5	98	680	18	6	6	D	3	5	7	13	60	36
15	F/37	C1 (anterior ring) -II odontoid fractures	Traffic accidents	7	9	C	3	128	430	12	6	6	D	2	3	5	9	33.3	36

Nerve injury was evaluated by Frankel grade [16]: C, useless motor function; D, useful motor function; E, recovery.

*ADI* atlanto-dental interval, *JOA* the Japanese Orthopaedic Association score, *M* male, *F* female.

### Surgical strategies

Posterior cervical pedicle screw fixation was performed on all patients as described in previous studies [11,13,15]. After general anesthesia, the patient was placed in a prone position and a midline incision was made to expose the C1 to C3 spinous process and lamina and then the atlantoaxial joint. The C1 pedicle screw was inclined by 0°–5° and placed at the intersection point which was formed at approximately 20 mm beside the midpoint of the atlantal posterior tubercle and 2 mm superior to the inferior edge of the posterior arch. The C1 pedicle screw should be perpendicularly inserted at the coronary plane and the tip of the screw was inclined to the head by 5° at the sagittal plane. The entry point for the C2 pedicle screw was located at the cranial and medial quadrant of the isthmus surface of C2. The C2 pedicle screw was directed 20° to 25° in a lateral-to-medial and cephalad trajectory. After tapping, a 3.5–4.0 mm multiaxial screw was inserted into the hole and the screw position was identified through C-arm fluoroscopy. When the atlantoaxial pedicle screw was entered, the cervical spine should be maintained at a mild supine and extension position so that when the connecting rod and the screw were locked by the retaining nut, the atlas pedicle screw would achieve a pulling effect by using the axial pedicle screw as a fulcrum point and, therefore, could gradually reset the dislocated atlas. If necessary, in order to achieve a satisfactory reduction of the atlantoaxial joint, it was also feasible to regulate the patient’s head position with the help of the assistant and fix it to the rod to maintain the alignment. Bone cortices of the arcus posterior atlantis, the axial lamina, and the spinous process were removed. The bone mass obtained from the posterior superior iliac spine was trimmed into the appropriate size of dovetail shape, the upper end of which was placed in the arcus posterior atlantis and the lower end of which was stuck between the axial lamina and the spinous process. The hollow part was filled with removed cancellous bones.

### Postoperative management and follow-up

After surgery, a drainage tube was placed for 24 to 48 h and antibiotics were routinely given for 1 to 3 days to prevent wound infection. Dexamethasone 10 mg/time once a day and mannitol 250 ml/time once a day were intravenously injected into the cases with neural symptoms for 3 days. After discharge, a plastic cervical gear was used for protection for 3 months. Follow-up visits were scheduled at every 3 months within 1 year as well as every 6 months one year later. Fracture healing was defined as trabecular bridging of the fracture and faint fracture line on the cervical spine X-ray [17,18]. Bone fusion criteria were less than 2° of movement between the spinous processes on flexion-extension lateral radiographs [19]. Movement of ≥2° on flexion/extension radiographs was regarded as a pseudarthrosis [20]. If pseudarthrosis could not be identified or excluded, CT scan was performed to evaluate about fusion. The Japanese Orthopaedic Association (JOA) scoring system for cervical myelopathy [21] was used to evaluate the treatment effects at 12 months after surgery compared with before operation. Recovery rate was calculated by the following formula: (postoperative score − preoperative score)/(17[full score] − preoperative score) × 100%. Recovery rates were graded as follows: >75%, excellent; 50% to 75%, good; 25% to 50%, fair; <25%, poor [22].

### Statistical methods

All data were expressed as mean ± SD and analyzed with Microsoft Excel 2010 (Microsoft, Redmond, Washington) and SPSS10.0 software (SPSS Inc., Chicago, USA). The difference between preoperative and postoperative JOA score was analyzed by paired *t*-test. Statistical significance was set at *P* <0.05.

## Results

**Figure 1:**
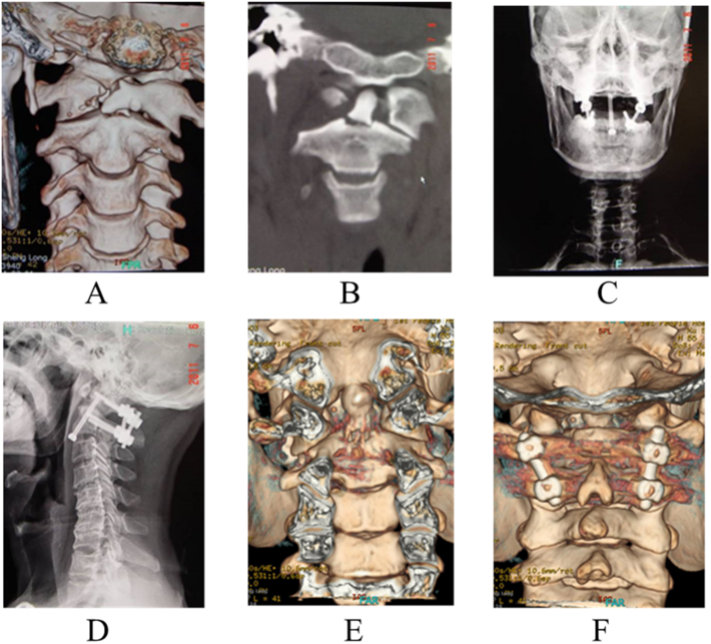
**A 36**-**year-old male patient was admitted to our hospital due to neck pain and restricted neck motion as well as incomplete limbs paralysis from traffic accidents.** Cervical spine 3D-CT scanning **(A)** and CT scanning in the coronal plane **(B)** showed that this patient suffered Jefferson combined with type II odontoid fractures. The patient underwent the posterior atlantoaxial pedicle screw internal fixation under general anesthesia **(C, D)**. At 12 months of follow-up, the 3D-CT scanning showed that the fracture was healed and the graft was fused **(E, F)**.

**Figure 2:**
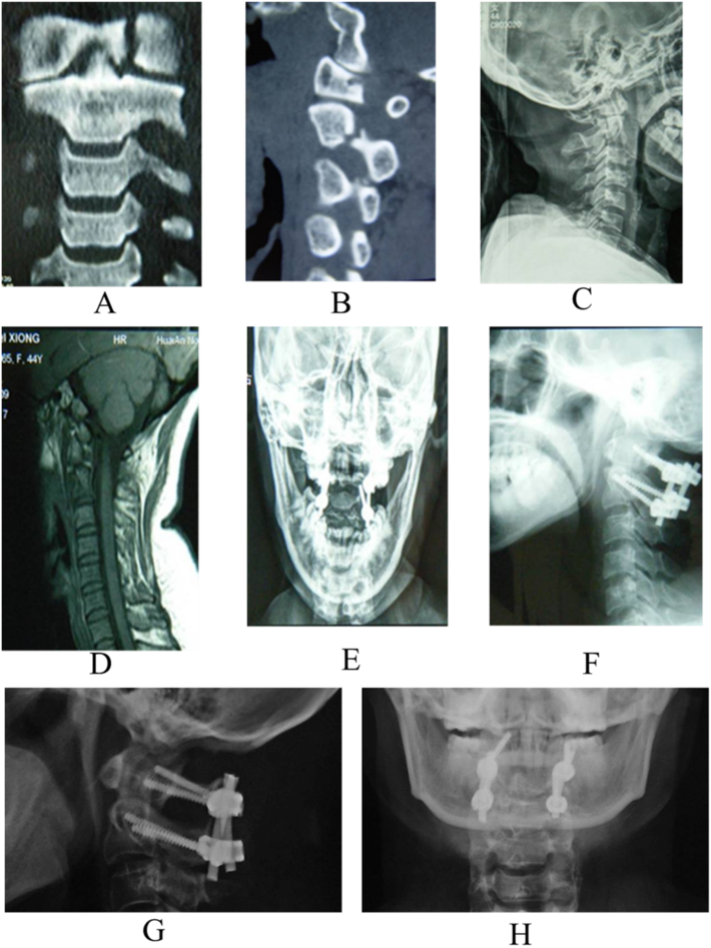
**A 47**-**year-old male patient was admitted to our hospital due to neck pain and restricted neck motion for 2 h from falling from a height.** Preoperative three-dimensional CT scanning **(A)**, X-ray **(B)**, and MRI **(C)** showed that anterior ring and hangman’s fracture. He underwent posterior atlantoaxial pedicle screw internal fixation. Postoperative anteroposterior **(E)** and lateral **(F)** X-ray showed that the atlantoaxial fracture was fixed well by the posterior atlantoaxial pedicle screw. At 9 months of follow-up, the anteroposterior **(G)** and lateral **(H)** X-ray showed that the fracture was healed well.

All the cases were followed up for a minimum of 12 months and a maximum of 58 months (mean, 36.5 ± 14.2 months). The mean operative time, blood loss, and hospital stays were 108.9 ± 25.8 min, 508.0 ± 209.6 ml, and 13.3 ± 2.0 days. The X-ray scan showed that the fracture healing and graft fusion were obtained in all patients within 9 months, with an average of 6.1 ± 1.7 and 6.8 ± 1.4 months. The ADI or C2-C3 angulation was reduced to ≤5 mm or ≤11°. No serious complications were observed postoperatively, such as internal fixation loosening, extrusion or breakage, secondary vertebral artery or nerve damage, nonunion, wound infection, and so on. JOA score was significantly improved from 7.3 ± 1.1 preoperatively to 15.7 ± 2.1 at 12 months after operation (*P* <0.001), with 88.1 ± 18.3% recovery rate and 93.3% excellent and good rate. The neurological situation was improved in all patients by at least 1 grade in the Frankel scale (see Table 1). The typical cases are shown in Figures 1 and 2.

## Discussion

Usually, complex atlantoaxial fractures frequently occur in elderly populations due to the mechanism of falling down. However, increasing motor vehicle accidents lead to the presence of complex atlantoaxial fractures in young adults recently. In this study, most of complex atlantoaxial fractures result from trauma in traffic accidents or falling from a height in young adults (<40 years).

Several surgery approaches have been attempted to treat complex atlantoaxial fractures, including Gallie wire [5], Brooks wire [6], interlaminar clamps [7], and transarticular screw [8-10]. The Brooks and Gallie wire techniques are easier to perform, but they require the use of an adjunctive halo vest to immobilize the fracture. It is reported that the nonunion rates reached to 33% even if the adjunctive halo vest was used [5]. Besides, wires are commonly inserted into the spinal canal, which leads to the patient being put at high risk for perioperative neurologic complications [23]. Subsequently, the transarticular screws are introduced and the transarticular screw is biomechanically considered more rigid (two-point fixation) than wiring (one-point fixation) and yields a higher fusion rate (>90%) [10,24,25]. However, the transarticular screw procedure is technically demanding due to the following two reasons: this technique requires reduction of atlantoaxial complex before screw placement; sufficient space must be available in the pedicle to avoid vertebral artery injury during placement of the screw. Thus, the vertebral artery injury complication may be easily induced [26]. Furthermore, the C1 lateral mass and C2 pedicle screws were put forward, which produced the similar results for stabilization of the atlantoaxial complex with a transarticular screw, but with a more superior radiological outcome [27]. Although Yoshida et al. reported that the C2-pedicle screw placement may have nearly the same anatomic risk of vertebral artery injury as the transarticular screw placement [28], preoperative three-dimensional evaluation and intra-operative fluoroscopy guidance may be useful for accurate placement of screws, thus reducing the incidence of vertebral artery injury. This was confirmed in a recent study in which CT angiography or magnetic resonance angiography was used to evaluate potential vertebral artery injury after surgery but none was found [29]. In this study, we also attempted to reduce the complex atlantoaxial fractures through posterior pedicle screws fixation and achieved the excellent outcomes, with the highest recovery rate up to 100% and without complication (e.g., internal fixation loosening, breakage, or vertebral artery injury). These results seemed to be in line with the study of Fu et al. [15] which focused on the complex atlas-axis fractures (C1-C3 odontoid fractures, seven cases; fracture-stable axis fractures, six cases; and C1 fracture-Hangman’s fractures, two cases), but which did not calculate the ADI and C2-C3 angulation.

However, there are potential limitations to our study. Firstly, as a retrospective study, patients were not randomly scheduled to a surgical procedure. The choice of surgery might be biased by the surgeons’ preference based on the preoperative condition of the patient. Secondly, because complex atlas-axis fractures rarely occur, it was difficult to obtain a sufficient number of patients. Thirdly, the follow-up period of some patients was not long. Therefore, future studies with a large sample size and longer-term monitoring need to be performed to verify our results.

## Conclusion

Posterior pedicle screw fixation is a reliable, effective, and minimally invasive procedure for patients suffering from complex atlantoaxial fractures.

## References

[1] Agrillo U, Mastronardi L (2006). Acute combination fracture of atlas and axis: “triple” anterior screw fixation in a 92-year-old man: technical note. Surg Neurol.

[2] Dickman CA, Hadley MN, Browner C, Sonntag VK (1989). Neurosurgical management of acute atlas-axis combination fractures: a review of 25 cases. J Neurosurg.

[3] Hadley MN (2002). Management of combination fractures of the atlas and axis in adults. Neurosurgery.

[4] Ryken TC, Hadley MN, Aarabi B, Dhall SS, Gelb DE, Hurlbert RJ, Rozzelle CJ, Theodore N, Walters BC (2013). Management of acute combination fractures of the atlas and axis in adults. Neurosurgery.

[5] Farey ID, Nadkarni S, Smith N (1999). Modified Gallie technique versus transarticular screw fixation in C1-C2 fusion. Clin Orthop Relat Res.

[6] Brooks AL, Jenkins E (1978). Atlanto-axial arthrodesis by the wedge compression method. J Bone Joint Surg.

[7] MOSKOVICH R, CROCKARD HA (1992). Atlantoaxial arthrodesis using interlaminar clamps: an improved technique. Spine.

[8] Stillerman CB, Wilson JA (1993). Atlanto-axial stabilization with posterior transarticular screw fixation: technical description and report of 22 cases. Neurosurgery.

[9] Dickman CA, Sonntag VK (1998). Posterior C1-C2 transarticular screw fixation for atlantoaxial arthrodesis. Neurosurgery.

[10] Haid RW, Subach BR, McLaughlin MR, Rodts GE, Wahlig JB (2001). C1-C2 transarticular screw fixation for atlantoaxial instability: a 6-year experience. Neurosurgery.

[11] Harms J, Melcher RP (2001). Posterior C1-C2 fusion with polyaxial screw and rod fixation. Spine.

[12] Nitising A, Jetjumnong C, Tisavipat N, Nantaaree S (2011). Posterior C1-C2 fusion using C1 lateral mass and C2 pars screw with rod fixation: techniques and outcomes. J Med Assoc Thai.

[13] Ma C, Wu J, Zhao M, Dai W, Wu D, Wang Z, Feng J, Liu C, Li Y, Zhao Q (2011). Treatment of upper cervical spine instability with posterior fusion plus atlantoaxial pedicle screw. Cell Biochem Biophys.

[14] Aryan HE, Newman CB, Nottmeier EW, Acosta FL, Wang VY, Ames CP (2008). Stabilization of the atlantoaxial complex via C-1 lateral mass and C-2 pedicle screw fixation in a multicenter clinical experience in 102 patients: modification of the Harms and Goel techniques. J Neurosurg Spine.

[15] Fu Y, Hu ZM, Huo HJ, Yang X, Hao T, Liu WL: **Improvement in JOA score of treatment for complex atlas-axis fractures.** *Pak J Med Sci* 2013, **29**(3):744. 10.12669/pjms.293.2995 PMC3809288 24353620

[16] Frankel HL, Hancock DO, Hyslop G, Melzak J, Michaelis LS, Ungar GH, Vernon JD, Walsh JJ (1969). The value of postural reduction in the initial management of closed injuries of the spine with paraplegia and tetraplegia. Paraplegia.

[17] Dijkman BG, Sprague S, Schemitsch EH, Bhandari M (2010). When is a fracture healed? Radiographic and clinical criteria revisited. J Orthop Trauma.

[18] Bhandari M, Chiavaras M, Ayeni O, Chakraverrty R, Parasu N, Choudur H, Bains S, Sprague S, Petrisor B (2013). Assessment of radiographic fracture healing in patients with operatively treated femoral neck fractures. J Orthop Trauma.

[19] Hacker RJ, Cauthen JC, Gilbert TJ, Griffith SL (2000). A prospective randomized multicenter clinical evaluation of an anterior cervical fusion cage. Spine.

[20] Yu S, Li F, Yan N, Yuan C, He S, Hou T: **Anterior fusion technique for multilevel cervical spondylotic myelopathy: a retrospective analysis of surgical outcome of patients with different number of levels fused.** *PloS one* 2014, **9**(3):e91329. 10.1371/journal.pone.0091329 PMC3949986 24618678

[21] Takagishi N, Nobuhara K, Fukuda H, Matsuzaki A, Mikasa M, Yamamoto R: **Shoulder evaluation sheet.** *J Jpn Orthop Assoc* 1987, **61:**623.

[22] Kawaguchi Y, Matsui H, Ishihara H, Gejo R, Yasuda T (2000). Surgical outcome of cervical expansive laminoplasty in patients with diabetes mellitus. Spine.

[23] Smith MD, Phillips WA, Hensinger RN (1991). Complications of fusion to the upper cervical spine. Spine.

[24] Taggard DA, Kraut MA, Clark CR, Traynelis VC (2004). Case-control study comparing the efficacy of surgical techniques for C1-C2 arthrodesis. J Spinal Disord Tech.

[25] Bahadur R, Goyal T, Dhatt SS, Tripathy SK: **Transarticular screw fixation for atlantoaxial instability-modified Magerl’s technique in 38 patients.** *J Orthop Surg Res* 2010, **5:**87. 10.1186/1749-799X-5-87 PMC2995783 21092173

[26] Gluf WM, Schmidt MH, Apfelbaum RI (2005). Atlantoaxial transarticular screw fixation: a review of surgical indications, fusion rate, complications, and lessons learned in 191 adult patients. J Neurosurg Spine.

[27] Lee SH, Kim ES, Sung JK, Park YM, Eoh W (2010). Clinical and radiological comparison of treatment of atlantoaxial instability by posterior C1-C2 transarticular screw fixation or C1 lateral mass-C2 pedicle screw fixation. J Clin Neurosci.

[28] Yoshida M, Neo M, Fujibayashi S, Nakamura T (2006). Comparison of the anatomical risk for vertebral artery injury associated with the C2-pedicle screw and atlantoaxial transarticular screw. Spine.

[29] Wang S, Wang C, Wood KB, Yan M, Zhou H (2011). Radiographic evaluation of the technique for C1 lateral mass and C2 pedicle screw fixation in three hundred nineteen cases. Spine.

